# Brivaracetam and rufinamide combination increased seizure threshold and improved neurobehavioral deficits in corneal kindling model of epilepsy

**DOI:** 10.1002/ame2.12478

**Published:** 2024-10-22

**Authors:** Awais Sattar, Zohabia Rehman, Hammad Murtaza, Waseem Ashraf, Tanveer Ahmad, Faleh Alqahtani, Imran Imran

**Affiliations:** ^1^ Department of Pharmacology, Faculty of Pharmacy Bahauddin Zakariya University Multan Pakistan; ^2^ Institut pour l'Avancée des Biosciences, Centre de Recherche UGA/INSERM U1209/CNRS 5309 Université Grenoble Alpes Saint Martin d'Hères France; ^3^ Department of Pharmacology and Toxicology, College of Pharmacy King Saud University Riyadh Saudi Arabia

**Keywords:** brivaracetam, corneal kindling, epilepsy, neurobehavioral analyses, oxidative stress, rufinamide

## Abstract

**Background:**

Besides seizures, a myriad of overlapping neuropsychiatric and cognitive comorbidities occur in patients with epilepsy, which further debilitates their quality of life. This study provides an in‐depth characterization of the impact of brivaracetam and rufinamide individually and in combination at 10 and 20 mg/kg doses, respectively, on corneal kindling‐induced generalized seizures and behavioral alterations. Furthermore, observed convulsive frequency and behavioral changes were correlated to post‐kindling‐induced changes in the activity of markers of oxidative stress.

**Methods:**

Adult C57BL/6 mice were kindled via twice‐daily transcorneal 50‐Hz electrical stimulations (3 mA) for 3 s for 12 days until animals reached a fully kindled state. After the kindling procedure, animals were tested using a set of behavioral tests, and neurochemical alterations were assessed.

**Results:**

Corneal‐kindled animals exhibited intense generalized convulsions, altered behavioral phenotypes typified by positive symptoms (hyperlocomotion), negative symptoms (anxiety and anhedonia), and deficits in semantic and working memory. BRV 10 + RFM 20 dual regime increased convulsive threshold and propensity toward the start of stage 4–5 seizures and improved phenotypical deficits, that is, anxiety, depression, and memory impairments. Moreover, this combination therapy mitigated kindling‐induced redox impairments as evidenced by reduced malondialdehyde and acetylcholinesterase levels and increased glutathione antioxidant activity in the brain of animals subjected to repetitive brain insult.

**Conclusion:**

Based on our outcomes, this dual therapy provides supporting evidence in alleviating epilepsy‐induced neurobehavioral comorbidities and changes in redox homeostasis.

## Graphical abstract

Corneal kindling might be a suitable etiological operative paradigm to characterize the potential impact of novel multimodal network therapeutics in managing interictal comorbidities of epilepsy. Brivaracetam (BRV) and rufinamide (RFM) combination therapy impeded kindling‐induced excitatory drive and seizure susceptibility compared to monotherapy. Furthermore, BRV and RFM combination therapy alleviated the progression of kindling‐associated psychiatric ailments typified by positive symptoms, negative symptoms, and cognitive decline. BRV and RFM combination therapy mitigated kindling‐induced alterations in redox balance. Our outcomes revealed that BRV and RFM combination therapy can provide translatable clinical benefits for patients with generalized epileptic disorder.

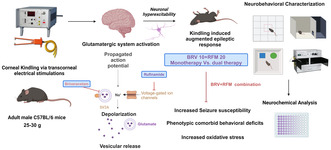

## 1 INTRODUCTION

Epilepsy is a complex spectrum disorder that entails more than just seizures. This multifactorial and multifaceted disabling disorder is characterized by enhanced excitatory synaptic composition and efficacy, leading to chronic network remodeling and spontaneous ictal events that are highly unpredictable in frequency from individual to individual.^1^ Nearly 30% of over 70 million epileptic patients globally have or can develop resistance, adverse reactions, and drug interactions to the current arsenal of antiseizure medications (ASM).^2^ Pharmacoresistant epilepsy contributes to reduced life expectancy, stigmatization, social discrimination, psychiatric multimorbidities, and a pulsating socioeconomic burden for public healthcare systems. Therefore, over the past decade, the intricate relationship between epileptogenesis, its diverse electro‐clinical manifestations, and associated behavioral dysfunction has been a research hotspot for developing new empirical investigational multimodal network therapies that can impede these underlying pathologies in patients afflicted with epilepsy.^3^ Moreover, clinical evidence postulates that inappropriate ASM monotherapy during the embryonic phase of the disease can affect future pharmacosensitivity, development of acquired‐epilepsy‐induced structural alterations, and comorbidities.^4^ Therefore, in the seemingly increasing armamentarium of the new second‐ and third‐ASM eras, rationally tailored polytherapy or dual therapy has gained more relevance as newer drugs have different and potentially synergistic mechanisms of action as well as better safety, efficacy, and tolerability compared to first‐generation ASMs.^5^

Historical screening of investigational candidate compound libraries relies on a diverse set of preclinical models of evoked seizures, and epilepsy as a single paradigm offers less relevance as an operative platform to predict the in vivo antiseizure efficacy of every new ASM. Unlike the acute seizure models in neurologically intact naive rodents, such as the maximal electroconvulsive shock test, subcutaneous pentylenetetrazole test, or more recently the 6‐Hz test, chronic kindling models are more relevant etiological platforms to reminiscent clinical epilepsy.^6^

Kindling can be defined as seizure‐induced neural plasticity, resulting in a progressive escalation in network‐driven seizure susceptibility and seizure‐induced behavioral characteristics in response to repeated subthreshold electrical or chemical triggers.^7^ Morphologically, kindling results in the increased excitatory drive via highly efficacious perforated synapses within the kindled brain, whereas at the molecular level, it results in long‐lasting glutamatergic storming, irregularities in GABAergic inhibition, perturbations in gene expression, and abnormal protein synthesis.^8^ In‐depth stimulation of deep brain structures via unilaterally implanted electrodes is a more resource‐intensive and time‐consuming model and requires electroencephalographic monitoring to investigate epileptogenesis and its interictal comorbidities. In contrast, corneal kindling with twice‐daily stimulations via transcorneal electrodes is a noninvasive and cost‐effective variant that helps to decipher translatable clinical benefits to patients with particular seizure profiles.^9^ However, sometimes the lack of electrographic recordings in these models is unfortunate because parallel quantification of behavioral manifestations and electrographic seizures offers a higher level of robustness and sensitivity in the management of epilepsy.

To date, for the newly established third‐generation ASMs, limited preclinical data are available on polytherapy with dose‐dependent additive effects and sophisticated approaches that can be exploited to halt chronic network excitability as well as seizure‐induced somatic and behavioral dysfunctions clinically in patients with diverse etiologies. Brivaracetam (BRV) is a new antiepileptic drug (4‐*n*‐propyl analog of levetiracetam) approved for partial‐onset seizures with a markedly higher selective affinity for synaptic vesicle glycoprotein (SV2A) in the brain. It alters neurotransmitter release by hoarding secretory vesicles, and an increased propensity for seizures has been observed in SV2A‐deficient animals.^10^ Rufinamide (RFM) (CGP 33101; 1‐([2,6‐difluorophenyl]methyl)‐1hydro‐1,2,3‐triazole‐4 carboxamide) is a triazole derivative used as an add‐on therapy for patients with Lennox–Gastaut syndrome (LGS). It modulates the activity of voltage‐gated sodium channels (VGSC) by prolonging the inactive state (slow recovery), thus stabilizing membranes and preventing repetitive high‐frequency action potentials, leading to neuronal bursting.^11^ Sodium channels play a key role in the maintenance of normal electrophysiological neuronal events, resting potential, and abnormal neuronal firing; therefore, inhibition of VGSCs will likely affect different seizure types.

Our present research contribution is the in‐depth characterization of the synergistic impact of combination therapy with BRV and RFM in corneal‐kindled mice. We aimed to decipher the enhanced protective potential of multimodal drug therapy versus monotherapy against seizure recurrence, behavioral alterations that congregate into positive symptoms (hyperactivity), negative symptoms (anxiety and depression), and cognitive deficits. Furthermore, we studied a link between neurochemical alterations, such as oxidative stress, kindling acquisition, and the development of secondary complications of epilepsy. Due to the efficiency of the corneal kindling model, the outcomes of this study provide supportive evidence for the efficacy of BRV + RFM dual therapy in controlling epilepsy‐induced comorbidities and altered redox balance.

## 2 MATERIALS AND METHODS

### 2.1 Animals, randomization, ethics, and experimenter blinding

Sixty adult C57BL/6 males weighing 25–30 g were bred at the animal house of the Faculty of Pharmacy, Bahauddin Zakariya University, Multan, Pakistan, and were housed in groups of five per cage at a constant temperature of 23–25°C and a 12‐light–dark cycle; the animals were provided standard rodent food pellets and tap water ad libitum. Experimenters were blinded by a third person who identified the animals from the ear tag and then simply randomized in blocks of 10 for six groups as described in the animal grouping with no significant difference in body weight and age.

### 2.2 Drugs and chemicals

BRV and RFM were obtained from Hilton Pharmaceuticals, Karachi. Phenytoin (PTN) was obtained from Atco Laboratories Ltd. (Pakistan) in a commercially available ready‐to‐use formulation (Epigran injection: 250 mg/5 mL), and 10 mg/kg was used. BRV was dissolved in distilled water, whereas RFM was suspended in 1% Tween 80 and used alone and in combination at 10 and 20 mg/kg doses, respectively. All drugs were freshly prepared daily and administered intraperitoneally. Information regarding dose and solubility was obtained from the previously published scientific literature.^12^, ^13^

### 2.3 Animal grouping and treatments

A total of 60 adult mice were randomly divided into six groups (*n* = 10 per group). Group 1: for the negative control, mock‐stimulated mice received 0.5 mL of 0.9% of normal saline once daily for 12 days followed by a sham or simulated stimulation of the cornea, mimicking the protocol used for corneal kindling but without the shock delivery, seizure induction, and subsequent epileptogenesis to decipher the impact of stress and animal handling in subsequent behavioral characterization. Group 2: the corneal kindling group received only electrical stimulations (50 Hz, 3 mA for 3 s) twice daily with an interval of 6 h between both corneal stimulations over the period of 12 days without any therapeutic intervention. Group 3: PTN 10 serves as a standard positive control (pretreated with PTN at 10 mg/kg + transcorneal electrical stimulations twice daily for 12 days). Group 4: BRV 10, animals were administered with a once‐daily dose of BRV as a standalone treatment at a dose of 10 mg/kg + transcorneal electrical stimulations twice daily for 12 days. Group 5: RFM 20, animals received a once‐daily dose of RFM at 20 mg/kg + transcorneal electrical stimulations twice daily for 12 days. Group 6: BRV 10 + RFM 20 (animals pretreated with a combination regime of BRV at a dose of 10 mg/kg and RFM at a dose of 20 mg/kg once daily + transcorneal electrical stimulations twice daily for 12 days).

### 2.4 Corneal kindling procedure

A rodent shocker system (Hugo Sachs Elektronik Harvard Apparatus, type: 221, serial no.: 08080, Germany) was used to deliver twice‐daily transcorneal electrical stimulations using saline‐soaked copper electrodes for 12 days consecutively (50‐Hz pulse frequency, 3‐mA intensity, 3‐s duration, and an interlude of 6 h between both electrical stimulations). Alcaine 0.5% eye drops (proparacaine hydrochloride, Alcon, Novartis) was applied prior to the placement of transcorneal electrodes for shock delivery. Seizure severity was assessed per the modified Racine's scale: 0, no seizure activity; 1, eye blinking and facial automatisms; 2, head nodding, mastication, and severe facial clonus; 3, whole body myoclonus; 4, bilateral forelimb clonus and rearing; 5, bilateral forelimb clonus with rearing and falling; and 6, tonic hind‐limb extension.^14^ By the 12th day, animals exhibiting consecutive stage 3 or higher‐grade seizures from Days 10 to 11 with twice‐daily transcorneal electrical stimulations were categorized as fully kindled per Matagne and Klitgaard who published a plateau in seizure severity after 10–12 days as further electrical stimulations failed to augment epileptic response in mice subjected to corneal kindling.^15^

**FIGURE 1:**
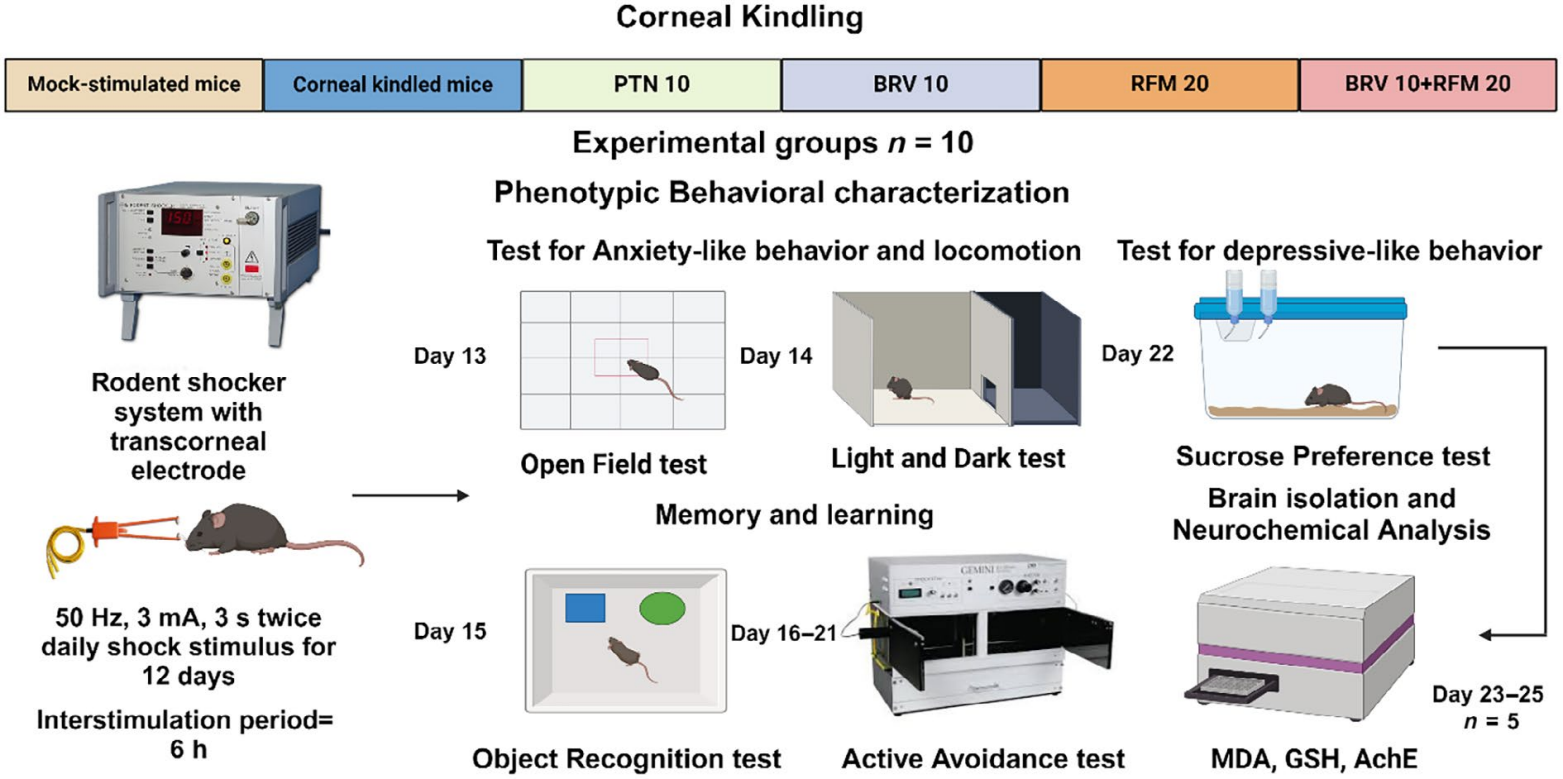
Experimental timeline for corneal kindling, behavioral characterization, and neurochemical analyses to quantify the impact of BRV 10 + RFM 20 dual therapy on kindling progression and associated psychiatric ailments.

Immediately after corneal kindling, animals were screened for phenotypic behavioral deficits in a fixed time window of 8:00 a.m.–5:00 p.m. by video monitoring in a series of least to most aversive tests. All neurobehavioral analyses were performed in the noiseless and uncrowded isolated facility by the experimenter blinded to current treatment protocols, and the animals were acclimatized for 1.5–2 h before commencement of any behavioral test. Subsequent to the behavioral assessment, brains were harvested to interpret kindling‐induced changes in markers of oxidative stress and its direct correlation to enhanced seizure susceptibility along with the potential beneficial impact of dual therapy of BRV 10 + RFM 20, as shown in Figure 1.

### 2.5 Open field test

Evaluation of spontaneous locomotive activity, exploration trajectories, rearing, and stereotyped movements are beneficial to decipher the impact of therapeutic interventions on the central nervous system as some drugs can induce sedation or motor impairment. On the 13th day, by the end of the corneal kindling protocol, each acclimatized animal was allowed to investigate a square box (45 × 45 × 20 cm, Panlab Harvard Apparatus IR Actimeter, Spain) provided with one upper frame to assess vertical movements and one lower frame to detect horizontal movements for 5 min. For precise subject detection, each square frame was provided with 16 × 16 infrared beams (photocell system) and an independent control unit. To examine hyperlocomotion, the number of vertical movements was noted in corneal‐kindled and treated rats. Furthermore, the whole experiment was recorded using a Logitech webcam to decipher the anxiolytic impact of dual BRV 10 + RFM dual therapy, and prerecorded videos were analyzed using the Any‐maze video tracking software (version 7.1, full licensed, Stoeltings Co., USA). The square frame was segregated into two zones, the central and peripheral, and animal's inclination toward the central arena was examined by observing the number of visits and exploration time in that particular zone.^16^

### 2.6 Light‐and‐dark test

The paradigm is based on the innate tendency of animals to show predilection toward the enclosed darker zone over an open and brightly lit aversive compartment of the light‐and‐dark (L/D) transition box.^17^ To estimate anxiety‐like behavior, animals were introduced into the illuminated compartment (21 × 21 × 25 cm), with black acrylic inserts used to segregate the illuminated and dark zones (20 × 40 × 40 cm) bridged by a small opening (7 × 6 cm). Animal's exploration time and number of visits were noted for the aversive illuminated compartment for 5 min to demonstrate variance in anxiety‐like behavior between kindled and treated animals.

### 2.7 Novel object recognition test

The object recognition test is a widely used platform to detect various phenotypic aspects of learning and cognitive deficits in experimental models of neurological disorders and involved two phases, namely acquisition and trial. During training, mice were permitted to investigate two identical objects for 5 min in the square box (40 × 40 cm), with walls high enough to discourage the animal's escape from the box (38 cm). During the trial session, one of the previously known object was shuffled with an unknown novel object, and the animal was allowed to explore for another 5 min after a retention interval of 1 h to access short‐term working memory.^18^ As mice have an innate inclination toward novelty, animals with intact short‐term recognition memory will likely tend to spend more time with the unfamiliar object rather than the familiar one. The object recognition index was calculated using the following formula:

$$\begin{array}{c} \text{Recognition index}=\left(\text{time with novel object}—\text{time with familiar object}\right)\\ /\left(\text{time with novel object}+\text{time with familiar object}\right). \end{array}$$

### 2.8 Active avoidance test

A Gemini two‐way shuttle box (Gemini, San Diego Instruments, USA) was used to assess long‐term spatial cue‐associated learning and memory retention in corneal‐kindled and BRV 10 + RFM 20‐treated rats. It consisted of two parallel compartments (24 × 46 × 20 cm) provided with an automatic guillotine sliding door, nonheating LED lights, and stainless steel grid floor (2.5 mm in diameter) and spaced ~10 mm apart. Additionally, a removable tray beneath the grid floor with a 2‐cm‐depth bedding was placed. The apparatus was cleaned each day and after each session with 70% isopropyl alcohol to remove feces, urine, or any possible odor clues that can impact the upcoming session or modulate the behavior of the next mouse in individual testing sessions. Gemini's software (Gemini) was used for automation and scoring of the active avoidance test.

Mice were gently placed in one of the compartments of the shuttle box after the 1‐h acclimation period in the testing facility and allowed to freely explore both chambers for 300 s. To examine active avoidance behavior in mice, after acclimation conditioned stimulus (CS), that is, houselights paired with an auditory tone (50 db, 2000 Hz), was employed for 10 s in the unoccupied chamber followed by the delivery of foot shock stimulus (unconditioned stimulus) of current intensity of 0.5 mA for 2 s in the occupied dark chamber as rodents have natural preference for a dark compartment, but after shock delivery they spontaneously moved toward the illuminated compartment until they learn to avoid the shock. Each animal was subjected to 30 trials per day for 6 days in which animals learned to escape the dark compartment upon presentation of CS to avoid shock stimulus. Three possible outcomes were typically observed with active avoidance: 1, mouse escapes into the safe compartment before the delivery of foot shock categorized as avoided response; 2, mice cross the adjacent compartment during the delivery shock stimulus categorized as escaped; and 3, mice did not shuttle to another chamber categorized as no response. An intertrial interval (ITI) of 20 s was given in which both CS and shock stimulus were turned off and the animal could move freely between both sections of the shuttle box until ITI elapsed, after which Gemini software automatically started a new trial. For 6 days, before the commencement of the trial, mice were introduced into an alternate chamber from the one that was previously used for shock delivery. Animal's mean % shock avoidance per trial per day was calculated to assess cue‐associated learning and memory consolidation.^19^

### 2.9 Sucrose preference test

Sucrose preference test (SPT) is a reliable paradigm to assess anhedonia or impaired reward‐based behavior, a characteristic of depression‐like behavior in rodents. After 12–16 h of fasting, mice were presented with a two bottles, and they had to choose one; one was filled with tap water and the other with 1% sweet sucrose solution^20^; percentage of sucrose preference was computed after an interval of 24 h using the following formula:

$$\begin{array}{c} \%\mathrm{SPT}=\text{sucrose solution intake}\;\left(\mathrm{mL}\right)/\text{sucrose solution intake}\;\left(\mathrm{mL}\right)\\ +\text{water intake}\;\left(\mathrm{mL}\right)\times 100. \end{array}$$

### 2.10 Brain isolation for neurochemical analysis

After the completion of neurobehavioral testing, mice were decapitated (*n* = 5), and the brains were isolated and individually homogenized in phosphate‐buffered saline solution (pH 7.4, Solarbio, Life Sciences) and then centrifuged for 10 min at 12000*g* at a controlled and constant temperature of 4°C.^21^ The pellet was discarded, and the clear supernatant was stored at −40°C to assess the neurochemical changes and preventive impact of dual therapy of BRV 10 + RFM 20 in corneal‐kindled mice. Protein was quantified for each brain sample using Lowry's method.^22^

### 2.11 Malondialdehyde

To evaluate corneal‐kindling‐induced lipid peroxidation, malondialdehyde (MDA) levels were analyzed calorimetrically. Briefly, 100 μL of brain supernatant was added to thiobarbituric acid‐trichloroacetic acid (TBA–TCA) in an equal ratio of 1:1. The reaction mixture was heated in a water bath maintained at 100°C and then cooled and centrifuged at 10000*g* for 10 min at 4°C. Finally, the reading was taken in duplicate at 532 nm using a microplate reader (Spectramax 340 PC384 by Molecular Devices, CA, USA), and MDA levels were individually normalized with protein content for all mouse brains.^23^

### 2.12 Reduced glutathione

To analyze the antioxidant activity of glutathione (GSH), 200 μL of the homogenate was added to the 120 and 50 μL of TCA. The mixture was then centrifuged for 15 min at 3000 rpm, and then 20 μL of the supernatant was added to 170 μL of Tris–HCl buffer (0.4 M, pH 8.9) and 10 μL of 5,5′‐dithiobis(2‐nitrobenzoic acid) (DTNB) (0.1 M); sample readings were taken in duplicate at 412 nm.^24^

### 2.13 Acetylcholinesterase

To measure acetylcholinesterase (AChE) activity, 40 μL of brain homogenate was mixed with 138 μL of phosphate buffer and 20 μL of 0.01 M DTNB; the basal reading was noted at 412 nm; then 2 μL of acetylthiocholine iodide (Sigma–Aldrich) was added. The reading was taken at 412 nm for up to 20 min with a time interval of 2 min.^25^

### 2.14 Statistical analysis

GraphPad Prism (version 8) for Windows (GraphPad Software, San Diego, CA, USA) was used for statistical data evaluation. Prior to any data evaluation, data normality was assessed using Shapiro–Wilk and Kolmogorov–Smirnov tests. One‐way analysis of variance (ANOVA) and then post hoc Dunnett's multiple comparisons were used to detect variance between the kindled and treated animals. Repeated two‐way ANOVA and then Dunnett's multiple comparison were performed for seizure scoring, whereas repeated two‐way ANOVA and then Tukey's test were used for % mean avoidance. All data were presented as mean ± standard deviation. A probability (*p*) value of <0.05 was considered statistically significant.

## 3 RESULTS

### 3.1 Impact of BRV 10 and RFM 20 alone and in combination on corneal‐kindling‐evoked augmented epileptic response and seizure recurrence

**FIGURE 2:**
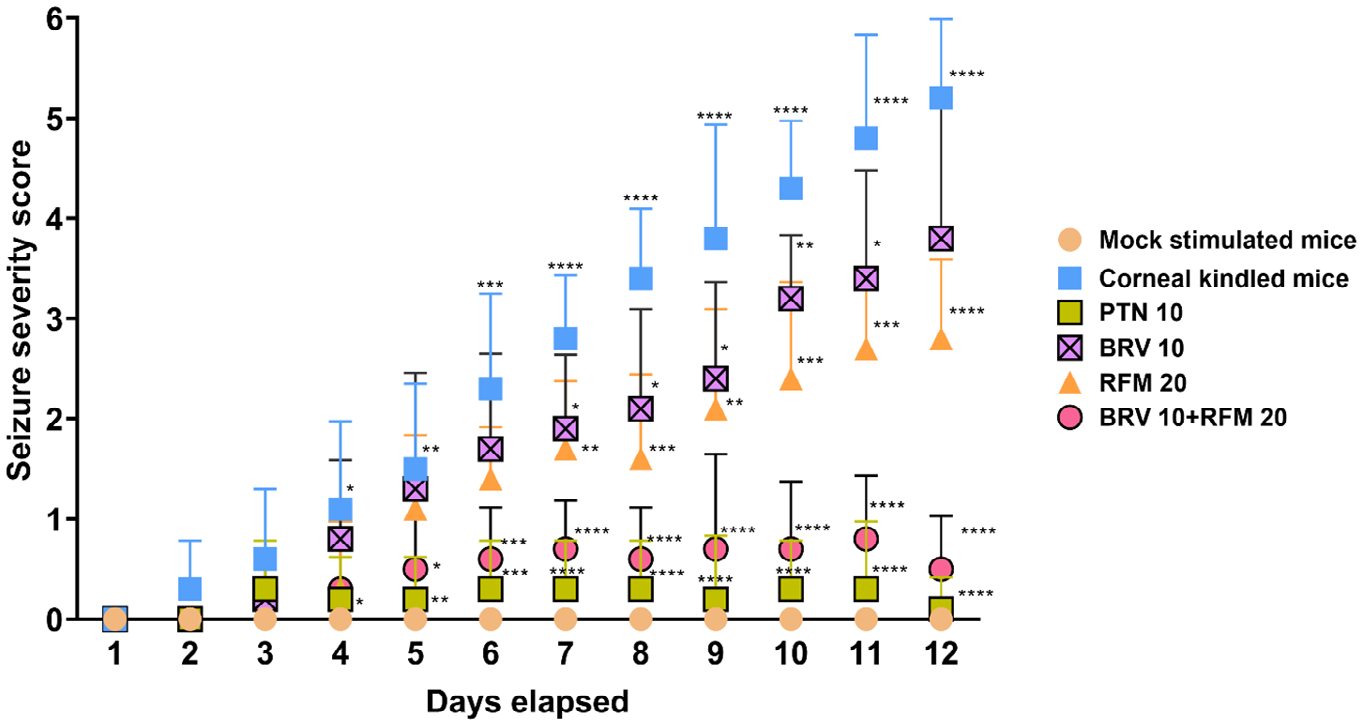
A graphical presentation of the impact of BRV 10 and RFM 20 alone and in combination on kindling progression as assessed using the modified Racine's scale. Mice were visually observed meticulously after every electrical stimulation twice a day for 12 days to demonstrate the potential benefits of dual therapy with diverse mechanisms of action on seizure intensity versus monotherapy as a standalone treatment in adult mice subjected to corneal kindling. Data are presented as mean ± SD (standard deviation). *p < 0.05, **p < 0.01, ***p < 0.001, and ****p < 0.0001 (*n* = 10).

Repetitive subconvulsive corneal shock (3 mA) for 3 s twice daily at an interval of 6 h between both electrical stimulations resulted in enhanced epileptic response, leading to high‐grade stage 5 seizures over a period of 12 days. Corneal kindling initially evoked partial seizures of stages 1 and 2, which gradually escalated to whole‐body clonus (stage 3) and eventually culminated into generalized convulsions of stages 5 and 6 by the end of the 12th day yielding fully kindled mice compared to mock‐stimulated animals (repeated‐measures two‐way ANOVA: *F*_5,594_ = 261.8, *p* < 0.0001). Monotherapy with BRV did not stop kindling progression as 7 of 10 animals were fully kindled by the 12th day similar to corneal‐kindled mice. Furthermore, in the RFM 20 group, the kindling acquisition rate was 30%, but most of the mice exhibited stage 3 convulsions by days 9–12. Reciprocally PTN 10 as a positive control significantly arrested the propagation of corneal kindling and the development of aberrant behavioral manifestations. Paradoxically, the combined therapy of BRV 10 + RFM 20 resulted in enhanced anticonvulsing effect and robust inhibition of seizure propagation. Dual therapy exhibited remarkable latency and increased convulsive threshold as all 10 of the corneal‐kindling‐challenged mice remained unaffected by high‐grade motor convulsions and therefore categorized as nonkindled. Behaviorally, only two animals exhibited stage 2 mild seizures from Days 10 to 12, and these findings were comparable with the mice receiving PTN 10 monotherapy (Figure 2).

### 3.2 Impact of BRV 10 and RFM 20 alone and in combination on kindling‐induced anxiety and hyperactivity assessed in open field test arena

**FIGURE 3:**
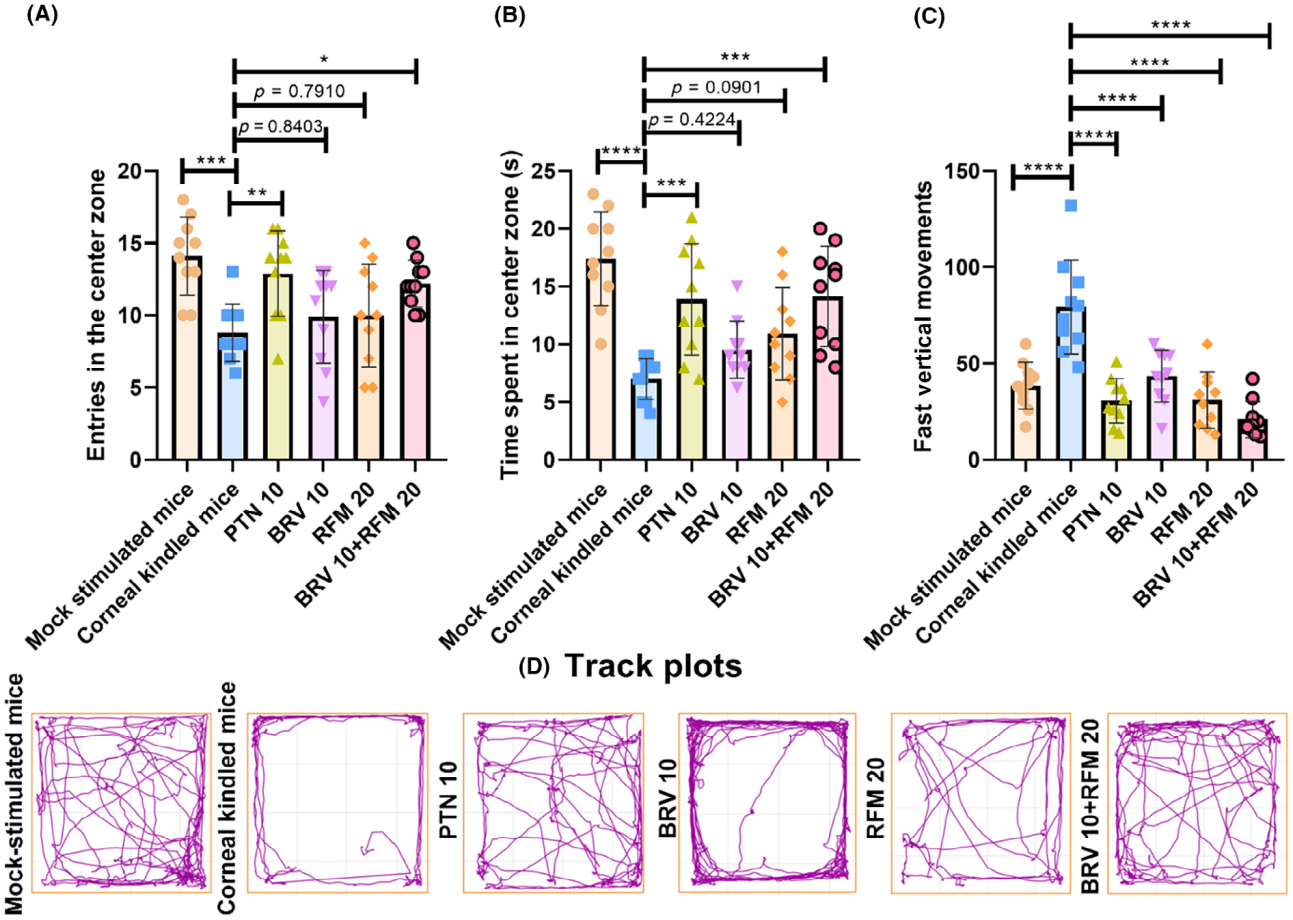
Assessment of post‐kindling exploratory activity to monitor the impact of mono and dual regimes of BRV 10 and RFM 20 on anxiety‐like behavior in mice. The animals spontaneously roamed the arena of the IR Actimeter for 5 min on the 13th day of study, and their (A) entries in the central arena, (B) investigation time in the central arena, and (C) fast rearing (vertical movements) were noted. (D) Track plots show the animal's inclination toward the central zone. Data are expressed as mean ± SD (standard deviation). *p < 0.05, **p < 0.01, ***p < 0.001, and ****p < 0.0001 (*n* = 10).

The animals' spontaneous locomotive activity in the open field arena was assessed to find their central zone preference by observing their number of visits (*F*_5,54_ = 5.562, *p* = 0.0003) and investigation time there (*F*_5,54_ = 9.995, *p* < 0.0001). The mock‐stimulated animals exhibited increased predilection toward the central zone with the increased number of visits (14.01 ± 2.68) and duration of stay (17.40 ± 4.06 s). However, corneal‐kindled mice wandered around the peripheries of the open arena and exhibited aversion toward the central zone, which indicates their anxiety‐like behavior with the reduced number of entries (8.80 ± 1.98) and investigation time (7.00 ± 1.76 s, *p* < 0.0001). This kindling‐prompted increased levels of anxiety were efficiently ameliorated by the combined regime of BRV 10 + RFM 20, as evidenced by their increased number of entries (12.20 ± 1.61, *p* = 0.0329) and duration of stay in the central zone (14.15 ± 4.31 s, *p* = 0.0004) as compared to disease controls. However, outcomes of monotherapy with both BRV 10 and RFM 20 remained nonsignificant compared to kindled mice, as shown in Figure 3A,B.

Furthermore, the spontaneous fast vertical locomotive activity was assessed during 5 min of the open field test (OFT), which was significantly increased in the corneal‐kindled group (79.30 ± 24.39) compared to their mock‐stimulated littermates (38.50 ± 12.23) and might indicate kindling‐induced hyperactivity. Kindled animals exhibited restlessness, leading to more frequent fast vertical movements such as rearing. Animals receiving monotherapy and dual therapy exhibited a decrease in fast‐rearing behavior, which was more pronounced in combination (20.90 ± 9.27, Figure 3C).

### 3.3 Anxiolytic characterization of BRV 10 and RFM 20 alone and in combination via L/D test

**FIGURE 4:**
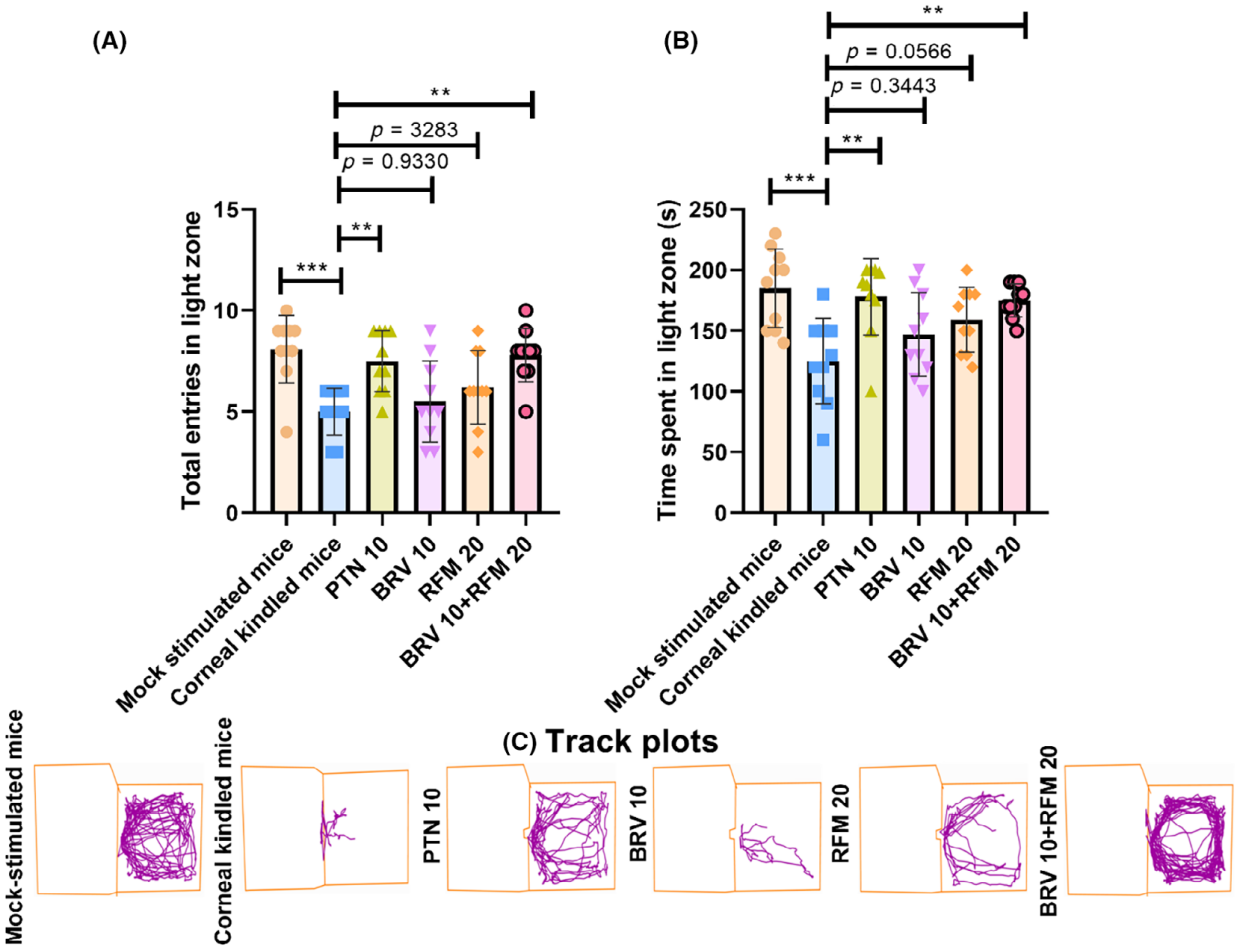
Assessment of the anxiolytic impact of BRV 10 and RFM 20 alone and in combination with corneal kindling evoked negative psychiatric alterations such as anxiety by testing them in an L/D (light‐and‐dark) box on the 13th day for 5 min to observe the (A) total entries in the illuminated zone, (B) exploration time in the illuminated zone, and (C) track plots exhibiting inclination of animals toward the brightly illuminated arena. Data are presented as mean ± SD (standard deviation). **p < 0.01 and ***p < 0.001 (*n* = 10).

The outcomes of the L/D box test further supported the findings of OFT, and anxiety‐like behavior was quantified by observing their number of visits (*F*_5,54_ = 6.513, *p* < 0.0001) and duration of stay (*F*_5,54_ = 5.707, *p* = 0.0003) in the aversive open illuminated zone. Corneal‐kindled mice exhibited significantly reduced inclination toward the illuminated compartment and tended to stay in the enclosed darker arena, as evidenced by their decreased number of entries (5.00 ± 1.15, *p* = 0.0003) and duration of stay (125.00 ± 35.04 s, *p* = 0.0002). However, sham controls exhibited a preference for a brightly illuminated open arena as most of the animals brazenly investigated the illuminated compartment with an increased number of visits (8.10 ± 1.66) and exploration time (185.00 ± 32.40 s) in that compartment. Furthermore, animals receiving dual therapy of BRV 10 + RFM 20 exhibited a prominent decrease in anxiety levels as they fearlessly explored the illuminated zone, resulting in an exceptional increase in the number of entries (7.80 ± 1.31, *p* = 0.0013) and duration of stay (175.00 ± 13.54 s, *p* = 0.0021) compared to disease control and monotherapy groups, as shown in Figure 4A,B.

### 3.4 Impact of BRV 10 and RFM alone and in combination on short‐term memory assessed via novel object recognition test

**FIGURE 5:**
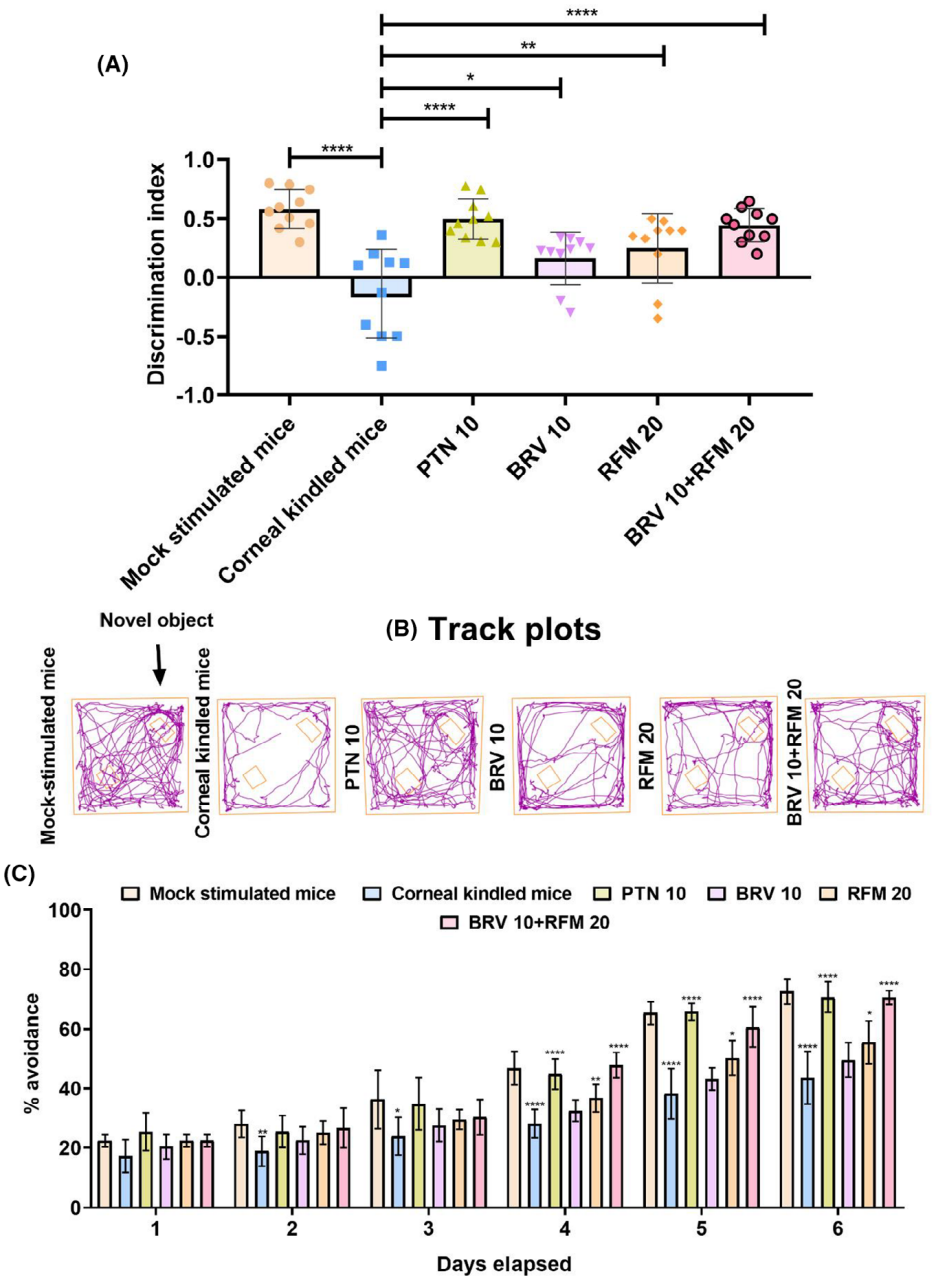
Assessment of the impact of BRV 10 and RFM 20 alone and in combination on post‐kindling‐induced cognitive dysfunction. On the 15th day, the animals were evaluated for their short‐term recognition memory in the NOR (novel object recognition) test as it is based on the animal's innate driven curiosity toward unknown novel objects. The object recognition index was examined to determine the animal's aptitude to discriminate between novel and previously familiar articles. Higher scores indicate good short‐term memory. (A) Discrimination index. From days 16 to 21 the animals were evaluated for their long‐term spatial memory in an active avoidance system where animals shuttled between two boxes to actively avoid shock in response to conditioned stimulus. The mean shock avoidance rate was calculated to quantify the impact of monotherapy versus dual therapy on post‐kindling acquired memory impairments. (B) Original tracking plots created by Any_Maze® software (C) Percentage avoidance. Data are presented as mean ± SD (standard deviation). *p < 0.05, **p < 0.01, and ****p < 0.0001 (*n* = 10) represent comparison between corneal‐kindled and treated mice.

The novel object recognition (NOR) test was conducted in two sessions, acquisition and trial, and the discrimination index was assessed to quantify the animals' learning and short‐term memory (*F*_5,54_ = 11.93, *p* < 0.0001). During the trial session, corneal‐kindled mice exhibited no remarkable inclination or exploratory interest toward the unknown novel object, as evidenced by their lower recognition score (−0.13 ± 0.37). In contrast, sham controls driven by their innate curiosity exhibited exceptional interest in the new object and spent most of their time sniffing the unknown object, which resulted in a higher discrimination index (0.58 ± 0.16). Furthermore, animals receiving BRV 10 and RFM 20 monotherapy also exhibited a slight significant increase in object recognition scores of 0.16 ± 0.22 and 0.24 ± 0.29, respectively, compared to corneal‐kindled mice. However, compared to disease controls, animals receiving dual therapy of BRV 10 + RFM 20 spent more time in exploring the unfamiliar object with a mean score of 0.44 ± 0.14, as shown in Figure 5A.

### 3.5 Impact of BRV 10 and RFM 20 on phenotypical deficits in long‐term working memory assessed via active avoidance task

The outcomes of the active avoidance task analyzed using repeated‐measures two‐way ANOVA showed a significant intergroup variance for the percentage of shock avoided (*F*_5,270_ = 79.39, *p* < 0.0001), further validating that corneal kindling might impact hippocampal‐driven learning and memory consolidation in mice. The corneal‐kindled mice demonstrated impaired cue‐associated learning, as evidenced by their reduced percentage of avoided trials by the end of the experiment. On the first day there was no statistically significant difference in percentage avoidance between mock‐stimulated and corneal‐kindled and between corneal‐kindled and treated mice as almost all the mice exhibited an average percentage avoidance of 20%–25%. On the second and third days, mock‐stimulated mice exhibited good memory, and their number of avoided trials increased significantly with *p* = 0.0049 and *p* = 0.0401, respectively, compared to disease controls, but no difference was observed in learning aptitude of corneal‐kindled and treated animals. On Day 4, the mice in the corneal‐kindled group exhibited a mean percentage avoidance of 28.21 ± 4.80 exhibiting poor memory consolidation compared to sham controls with a mean percentage avoidance of 46.80 ± 5.65, *p* < 0.0001. Outcomes in mice receiving BRV 10 remained nonsignificant (32.47 ± 3.59%, *p* = 0.2512), and RFM 20 as a standalone treatment also demonstrated a slight increase in percentage avoidance (36.72 ± 4.61, *p* = 0.0078) compared to corneal‐kindled mice. In contrast, BRV 10 + RFM 20 exhibited an increase in shock avoidance (47.87 ± 4.30%) with *p* < 0.0001. On Day 5, disease controls exhibited a shock avoidance rate of 38.23 ± 8.50, which was significantly higher in sham controls (65.24 ± 3.75%) with *p* < 0.0001. Mice supplemented with a combination therapy of BRV 10 and RFM 20 exhibited good cue‐associated learning with a mean percentage avoidance of 60.68 ± 6.72 with *p* < 0.0001, and RFM alone also exhibited an increase in shock avoidance rate (50.29 ± 5.91%, *p* = 0.0206) compared to disease controls. On the last day of the experimental trial, corneal‐kindled animals still exhibited impaired learning, as evidenced by their reduced mean shock avoidance rate (43.58 ± 8.85%) when compared to the mock‐stimulated negative control (72.47 ± 4.25%, *p* < 0.0001). Dual regime supplementation halted corneal‐kindling‐induced cognitive phenotypical deficits and resulted in good learning with an increased mean avoidance rate of 70.46 ± 2.38%, *p* < 0.0001, compared to mice with memory dysfunction in the corneal‐kindled group. RFM 20 monotherapy also attenuated kindling‐induced learning dysfunction to some extent as animals exhibited a mean avoidance rate of 55.53 ± 7.28%, *p* = 0.0407, by the end of the experimental trials. However, outcomes with BRV 10 remained statistically nonsignificant compared to disease controls, as shown in Figure 5C.

### 3.6 Impact of BRV 10 and RFM 20 alone and in combination on kindling‐induced depression‐like behavior in saccharin intake test

**FIGURE 6:**
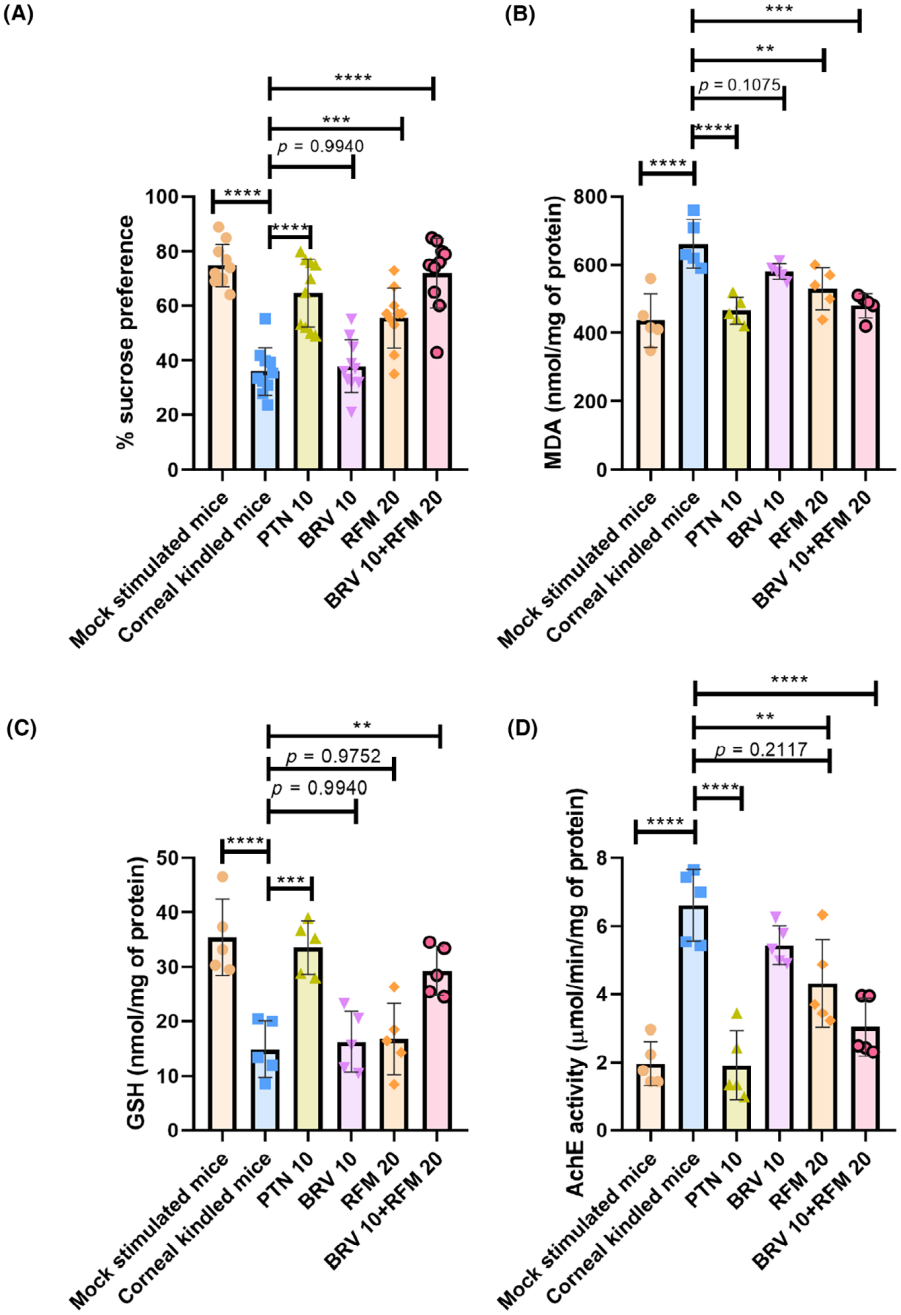
Assessment of the impact of BRV 10 and RFM 20 alone and in combination on depression‐like phenotype and markers of oxidative stress. On the 22nd day, a relative preference for sweetened sucrose solution was observed to examine kindling‐induced anhedonia. (A) Percentage of sucrose preference. (B–D) Impact of dual therapy on kindling‐induced redox impairments. On the 23rd day brains were obtained to study markers of oxidative stress in mock‐stimulated, corneal‐kindled, and combination‐treated mice. (B) MDA (malondialdehyde, nmol/mg of protein), (C) GSH (glutathione, nmol/mg of protein), and (D) AchE (acetylcholinesterase) activity (μmol/min/mg of protein). Data are expressed as mean ± SD (standard deviation). **p < 0.01, ***p < 0.001, and ****p < 0.0001 (*n* = 10 for SPT [sucrose preference test] and *n* = 5 for neurochemical assays).

A saccharin intake test was employed to characterize anhedonia in kindled versus treated mice (*F*_5,54_ = 25.15, *p* < 0.0001). Post hoc analysis showed that corneal‐kindled mice exhibited impairment in reward behavior as evidenced by their reduced sucrose preference (36.02 ± 8.81%) compared to sham controls (74.90 ± 7.83%) with *p* < 0.0001. Compared to disease controls, RFM monotherapy at a dose of 20 mg/kg resulted in an increased preference for sweet fluids (55.6 ± 10.92%, *p* = 0.0006) over tap water. Furthermore, dual therapy remarkably arrested the progression of kindling‐induced depression‐like behavior as animals exhibited enhanced inclination toward sweetened 1% sucrose solution over tap water (72.10 ± 12.86%, *p* < 0.0001) compared to corneal‐kindled mice. However, outcomes with BRV 10 monotherapy remained statistically nonsignificant, and the results were almost similar to those of the animals in the corneal‐kindled group, as shown in Figure 6A.

### 3.7 Impact of BRV 10 and RFM 20 alone and in combination on markers of oxidative stress

The marked variance was noted for MDA levels between diseased and treated mice (*F*_5,24_ = 11.51, *p* < 0.0001), as shown in Figure 6B. Post‐kindling, mice exhibited increased MDA levels (662.30 ± 70.54 nmol/mg of protein) compared to mock‐stimulated healthy brains (437.32 ± 77.55 nmol/mg of protein) with *p* < 0.0001. RFM 20 monotherapy decreased MDA levels to 530.04 ± 62.94 nmol/mg of protein with *p* = 0.0040 compared to the brains of corneal‐kindled mice. Compared to disease controls, the brains of animals supplemented with BRV 10 + RFM 20 dual regime demonstrated a prominent reduction in lipid peroxidation levels (480.22 ± 35.69 nmol/mg of protein, *p* = 0.0001).

Furthermore, the activity of antioxidant enzymes such as GSH was found to be significantly varied between disease controls and combination‐treated animals (*F*_5,24_ = 13.76, *p* < 0.0001). Corneal kindling induces persistent generalized convulsions that impact redox homeostasis in the brains of diseased animals as evidenced by reduced GSH levels (14.86 ± 5.17 nmol/mg of protein, *p* < 0.0001) compared with mock‐stimulated controls (35.39 ± 6.94 nmol/mg of protein). The combined regime significantly increased GSH activity (29.28 ± 4.57 nmol/mg of protein, *p* = 0.0023) in the brains of animals pretreated with this drug combination, with subsequent exposure to twice‐daily electrical stimulations for 12 days. Outcomes with monotherapy of both BRV 10 and RFM 20 remained nonsignificant compared to corneal‐kindled disease controls, as shown in Figure 6C.

Additionally, AchE levels were significantly different between all groups (*F*_5,24_ = 20.93, *p* < 0.0001). Diseased brains exhibited increased AchE activity (6.60 ± 1.05 μmol/min/mg of protein, *p* < 0.0001), which was significantly lower in mock‐stimulated mice (1.96 ± 0.64 μmol/min/mg of protein). Monotherapy with RFM 20 demonstrated a slight decrease in AchE levels compared to the brains of disease controls (4.30 ± 1.29 μmol/min/mg of protein, *p* = 0.0031). In contrast, the combination regime of BRV 10 + RFM 20 showed a significant reduction in AchE levels (3.02 ± 0.84 μmol/min/mg of protein) with *p* < 0.0001. BRV 10 monotherapy yielded nonsignificant results, and it did not exhibit any protection toward kindling instigated redox impairments (Figure 6D).

## 4 DISCUSSION

Together with intractable unprovoked seizures, a myriad of overlapping psychological and cognitive multimorbidities further deteriorate the quality of life in patients with epilepsy.^26^ Intriguingly, the deleterious alterations underlying the pathophysiology of these comorbidities are speculated to merge with upregulated network‐driven detrimental signaling pathways prompting neuronal reorganization and seizures. Moreover, the origin of psychiatric and cognitive disturbances has a positive correlation with pharmacoresistance to ASMs.^27^ Over the recent years, the United States National Institute of Mental Health approved a diagnostic outlook that suggests polythetic categorization of mental ailments in the terrains of multitudinous symptoms. Positive symptoms (endophenotypes of psychosis, i.e., hyperlocomotion), symptoms of negative valence system (anxiety, fear, panic, and social aversion), and cognitive impairments (deranged learning, focus, episodic, spatial, visual, and contextual fear memory) often accompany epilepsy and other neurological disorders and have been described in kindled, post‐status epilepticus and genetic preclinical experimental models of epilepsy.^28^, ^29^ Nonetheless, the key contributors to cognitive and behavioral disruptions in epilepsy are seizure activity, neuronal network reorganization, and resistance to ASMs.

In epilepsy treatment, monotherapy is the preferential choice in newly diagnosed epilepsy given similar safety, efficacy, and relatively superior tolerability to polytherapy.^30^ However in the case of failure of two monotherapy trials and recurrent seizures, the combination regime with multimodal network therapeutics that can produce synergism has gained much attention to control the propagation of ictal events, structural alterations, and coexisting comorbidities such as anxiety, depression, and memory disturbances. A current topic in epilepsy research is to recapitulate as many pharmacologic, behavioral, and heterogeneous clinical aspects of the enigmatic phenomenology of epileptogenesis in preclinical animal models. In the current study, we aimed to decipher the impact of BRV and RFM alone and in combination at 10 and 20 mg/kg, respectively, on seizure recurrence and kindling‐induced isomorphic clinical cognitive and psychic multimorbidities of epilepsy. Additionally, we studied whether these observed seizure episodes and behavioral perturbations could be linked to changes in redox homeostasis throughout the brain.

The present study used the experimental corneal kindling model to demonstrate the impact of BRV 10 and RFM 20 alone and in combination on the network‐driven propagation of the seizure activity, that is, from mild partial seizures to bilateral tonic–clonic convulsions (stage 4–5 convulsions). Compared to corneal‐kindled mice, the combinatorial regime demonstrated prominent protection from brain insults induced via twice‐daily transcorneal electrical stimulations for 12 days. BRV 10 as a single drug exhibited no significant protection from the kindling‐induced progression of epileptic seizures as seizures of high severity were noted from Days 6 to 12. RFM 20 alone initially exhibited mild partial seizure from days 1 to 8 but later exhibited a greater prevalence of stage 3 seizures, with further repetitive electrical stimulations from Days 9 to 12. However, marked propensity was observed toward the development of stage 4–5 grade seizures with dual therapy of BRV 10 and RFM 20, which might provide supporting evidence for potential translatable clinical benefits and use of network therapeutics for the robust management of seizure chronicity compared to monotherapy.

Psychiatric comorbidities (anxiety, depression, mania, panic disorder, and memory loss) and seizure chronicity have been considered to overlap for millennia. Approximately 20% of patients with intractable epilepsy suffer from anxiety, and symptoms may manifest as intrinsic aspects of seizure episodes and can occur as seizure prodromes (pre‐ictal), interictal, or post‐ictal anxiety.^31^ Anxiety disorder and epilepsy share some common underlying neurochemical aspects, that is, reduced GABA levels and serotonin receptor binding in particular, as GABAmimetic drugs can reduce both seizures and neuroticism.^32^ However, the neurobiological intertwined mechanisms that can trigger chronic anxiety in epilepsy can be aberrant neuroplasticity within deep brain structures; dysfunction of the hypothalamic–pituitary–adrenal axis; and changes in neurochemical pathways such as brain‐derived neurotrophic factor and others like genetic architecture, early‐life stress, or neurotrauma.^33^ Neurobehavioral characterization in corneal‐kindled mice exhibited a significant increase in anxiety levels compared to mock‐stimulated littermates. Furthermore, findings of OFT and L/D validated that administration of BRV 10 + RFM 20 did not impact the animal's spontaneous locomotive activity, and no central nervous system depressant effects were observed at this dose. BRV 10 as an SV2A glycoprotein ligand and RFM 20 through its impact on VGSCs affected kindling‐induced progression of comorbid anxiety disorder, and a significant reduction in anxiety‐like behavior was observed in mice treated with BRV 10 + RFM 20. Monotherapy did not yield any statistically significant results compared to diseased animals, and these outcomes are in line with the results of Farooq et al. who observed that 10 mg/kg of BRV in Pentylenetetrazole‐kindled BALB/c mice did not exhibit any prominent decrease in anxiety‐like behavior, as evident from the decreased number of entries and time spent in the illuminated compartment in the L/D transition test.^34^

Cognitive disruptions in epilepsy are a result of peculiar interactions among the etiologies of epileptogenesis, interictal discharges, seizures, and ASMs.^35^ Complex underpinning mechanisms underlying pathophysiogenesis between epilepsy and memory involve the periodic interictal epileptiform discharges and seizures potentially injuring neural networks that are key substrates for normal cognitive function and information processing. An alternative hypothesis is that the disruption of neural networks with subsequent morbidities is caused by the etiology of epilepsy, that is, traumatic injury, single gene mutations, and malformation in cortical development. Moreover, hippocampal sclerosis and a decrease in neural density are characteristic features of both partial and generalized epilepsy.^36^ A previous investigation by Operto et al. showed no negative impact of add‐on RFM therapy on LGS patients initially on 100–2400 mg/kg for 12 months.^37^ Moreover, Nygaard et al. have reported no memory impairment with BRV treatment in mice with Alzheimer's disease.^38^ Our study observed severe memory impairments in corneal‐kindled rats, which were prominently alleviated using BRV 10 + RFM 20 in both NOR and active avoidance tests, hinting at the possible modulation of SV2A expression with subsequent restoration of depressed long‐term potentiation and synaptic dysfunction. RFM, on the contrary, reduces glutamatergic storming and therefore prevents further neuronal loss and abnormal structural alterations leading to cognitive deficits.

Anhedonia in humans is directly correlated with social impairment and is a sign of depression.^39^ Neurotransmitter abnormalities, temporal lobe structural alterations, and prefrontal volume changes are the major contributors to the development of depression and may also trigger the kindling process of epileptic foci, increased seizure frequency, and intensified seizure predisposition in preclinical animal models of epilepsy.^40^ Moreover, the limbic–cortical‐striatal‐pallidal‐thalamic tract involved in major depressive disorders overlaps with brain regions affected by epilepsy.^41^ Toledo et al. published a prospective analysis report in which BRV treatment improves mood score with no psychiatric adverse effects in epileptic patients.^42^ Fava reported significant clinical improvement in depression and mood‐stabilizing impact of RFM at 800 mg/kg in a woman with bipolar disorder.^43^ Our outcomes revealed impaired reward behavior in kindled animals, which was significantly improved using combination therapy as mice exhibited greater preference for sweetened sucrose solution.

Oxidative stress and epilepsy have a bidirectional relationship. Hyperexcitability, the key neurophysiological feature of epilepsy, is accompanied by neuroinflammation, increased metabolic demand and production of reactive oxygen species, and disturbed endogenous antioxidant homeostasis, resulting in enhanced seizure susceptibility.^44^, ^45^ Some ASMs have antioxidant properties, which may add to their clinical and neuroprotective benefits. We correlated post‐kindling behavioral impairments with neurochemical alterations and observed positive associations of changes in neurochemical markers of oxidative stress and increased excitatory drive with cognitive deficits in corneal‐kindled rats. Our study observed a three‐ to fivefold increase in MDA and AchE levels and a decrease in GSH in the brains of diseased animals versus mock‐stimulated mice. BRV 10 + RFM 20 therapy efficiently mitigated post‐kindling‐induced redox balance alterations.

## 5 CONCLUSION

Overall, our neurobehavioral characterization demonstrated the efficiency of the corneal kindling animal model in recapitulating isomorphic clinical multimorbidities of epilepsy. In addition, we studied the impact of monotherapy and dual therapy with BRV 10 and RFM 20 on seizure susceptibility, chronicity, and associated behavioral dysfunction with post‐kindling changes in oxidative stress. Our outcomes revealed that BRV 10 + RFM 20 has an important role in corneal kindling and its neuropsychiatric comorbidities. This dual therapy remarkably alleviated the pathogenesis of generalized convulsions, arrested kindling progression, and mitigated anxiety and depression‐like behavior along with cognitive deficits. Furthermore, it modulated the altered redox balance, increasing the convulsive threshold and causing neuropsychological disruptions in pretreated mice subjected to corneal kindling. Corneal kindling may be used as a productive tool to probe and map the pathogenesis of network disorder in neuronal ensembles, and also to discover the efficacy of new drug combinations that presumably have not been previously characterized for generalized epilepsy and associated comorbidities.

## AUTHOR CONTRIBUTIONS

Experiments were performed by Awais Sattar, Zohabia Rehman, and Hammad Murtaza. Experiments were designed by Faleh Alqahtani, Imran Imran, and Waseem Ashraf. Awais Sattar, Zohabia Rehman, Hammad Murtaza, and Waseem Ashraf analyzed the data. Awais Sattar, Zohabia Rehman, Waseem Ashraf, and Imran Imran wrote the paper, with assistance from Tanveer Ahmad and Faleh Alqahtani in editing and revisions. Waseem Ashraf, Faleh Alqahtani, and Imran Imran supervised the work.

## FUNDING INFORMATION

The authors extend their appreciation to the Distinguished Scientist Fellowship program at King Saud University, Riyadh, Saudi Arabia, for funding this work through Research Supporting Project Number RSP2024R131.

## CONFLICT OF INTEREST STATEMENT

The authors declare no potential conflicts of interest.

## ETHICS STATEMENT

All animal experiments were performed per the national guidelines and policies on animal experimentation and were approved by the ethics committee for the utilization of laboratory animals of the Department of Pharmacology, BZU, Multan, Pakistan (Ethical Committee Letter no.: 10‐PHL‐S22; January 11, 2023). To ensure animal welfare, all in vivo tests were performed in accordance with the ARRIVE guidelines.
